# Information Needs of People With Limited Health Literacy Regarding a New “Opt-Out” Organ Donation System: A Qualitative Study in the Netherlands

**DOI:** 10.3389/ti.2022.10295

**Published:** 2022-03-17

**Authors:** Jany Rademakers, Marlon Rolink, Monique Heijmans

**Affiliations:** ^1^ Nivel, Netherlands Institute for Health Services Research, Utrecht, Netherlands; ^2^ Department of Family Medicine, CAPHRI (Care and Public Health Research Institute), Maastricht University, Maastricht, Netherlands; ^3^ Amsterdam UMC, Location AMC, Amsterdam, Netherlands

**Keywords:** organ donation, health literacy, information, communication, opt out

## Abstract

**Background:** In the Netherlands, new legislation on organ donation was implemented, based on a “opt-out” consent system, which means that all adults are presumed to consent for organ donation, unless they actively register their decision not to donate. A public information campaign preceded the law change. In the Netherlands, 29% of the population has limited health literacy (LHL). The aim of the study was to gain insight in the information needs of Dutch citizens with LHL regarding organ donation and the new legislation, as well as in their preferred information channels.

**Methods:** A qualitative study was performed; 30 people participated in four focus groups and six individual interviews. Transcripts were coded, interviews were thematically analysed.

**Results:** People with LHL need specific information to make an informed decision on organ donation. Relevant topics: 1) choice options, 2) eligibility, 3) role of partner and/or family, 4) impact on quality of care, and 5) process of organ donation. Information should be easy to understand.

**Conclusion:** Current standard materials are too difficult and abstract. People with LHL require personal support to tailor general information to their personal situation, and practical help to actually register their choice. Suggestions on how to improve information is provided.

## Graphical Abstract

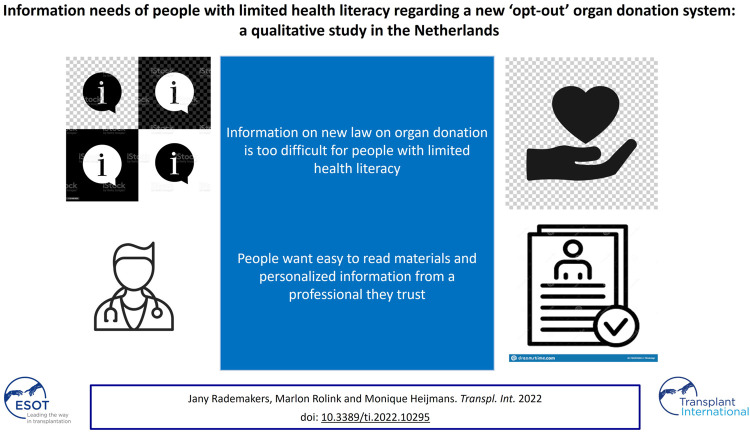

## Introduction

Globally, there is a shortage in organ donors to meet the demand for organ transplantation [1–3]. As a result, waiting lists of patients in need for organ or tissue transplantation are growing, and a substantial part of these patients die while on the list [1]. There are several pathways considered effective in increasing transplantation rates, one of which is the implementation of a legal system based on “presumed consent” or “opt-out” [1, 2]. The essential difference between an “opt-out” and an explicit consent or “opt in” system is that in the latter, citizens are not automatically considered organ and tissue donors unless they have actively registered as such. In an “opt-out” system, all citizens of 18 and older are presumed to be a donor, unless they choose to state otherwise. Several European countries have successfully implemented a legal ‘opt-out’ system: Austria, Belgium, Czech Republic, Croatia, Finland, France, Hungary, Italy, Latvia, Norway, Portugal, Slovakia, Slovenia, Spain, Sweden and the United Kingdom [3, 4].

In the Netherlands, a new Organ Donation Act based on “opt-out” came into force in 2020. Between September 2020 and March 2021, all adult Dutch citizens received a letter with information about the new Organ Donation Act and the request to enter their choice (online or through a paper form) in the Donor registry. In general, there are four choice options: 1) yes, I consent to being an organ donor, 2) no, I do not consent to being an organ door, 3) I want my partner or relatives to decide this after my death, 4) I want another (specified) person to decide this after my death. One can also exclude specified organs for donation. If -even after a reminder- people do not register their choice, they are automatically registered as an organ donor (option 1). Since the register was already in place, Dutch citizens could also have entered their choice earlier. In this case people were advised to check if their choice was still valid. In order to inform and prepare the general public, the Dutch government launched a national information campaign in 2019 through different channels (leaflets/brochures, advertorials, posters, television/radio commercials, short films, a website). On top of this national campaign, the Dutch Ministry of Health decided to develop and implement additional communication strategies for specific subgroups who might have different information needs and more difficulties in understanding and applying this information, f.e., people with cognitive impairments and citizens with limited health literacy (LHL).

Health literacy is the ability to access, understand, appraise and apply health information in the domains of healthcare, disease prevention, and health promotion [5]. It entails the capacity to read and write (functional health literacy), but also more advanced cognitive and social skills. In a recent review, three key elements of health literacy were discerned: 1) cognitive attributes (knowledge, functional skills, comprehension and understanding, appraisal and evaluation, critical thinking); 2) behavioural and operational attributes (seeking and accessing information, communication and interaction, application of information, citizenship); and 3) affective and conative attributes (self-awareness/self-reflection, self-control/self-regulation, self-efficacy, interest and motivation) [6]. Thus, health literacy is more than a cognitive skill or the “capacity to think.” Especially if an active role of people is required, the “capacity to act” is even more important [7]. Regarding the Organ Donation Act, people not only need the skills to access and understand the given information, but also to develop a personal opinion about the different choice options and to actually enter their choice in the register. Given the fact that 29% of the Dutch population has inadequate or limited health literacy [8], providing clear information on the Organ Donation Act that fits the needs of this group and motivates them to actively register their personal choice was considered as a challenge by the Dutch government [8]. At the time the national communication campaign in the Netherlands was launched, there still was lack of evidence on how to best inform citizens with LHL about organ donation in general and about an ‘opt-out’ system in particular, neither in the Netherlands nor in most other European countries that have implemented such legislation [3, 4]. Wales was the only country that had acknowledged the information needs of specific subpopulations (elderly, people with limited reading skills, lower educated citizens, black/ethnic minority groups) and tailored their information and channels accordingly [4, 9]. Earlier studies in different domains (e.g., cancer screening, screening for Down in pregnant women, health decisions in general) had already demonstrated that people with LHL have more difficulties with understanding and applying health information and making informed choices, and that they need specific and tailored information [10–16].

To get better insight in the information needs of Dutch citizens with LHL regarding the new Organ Donation Act as well as in their preferred information channels, the Dutch Ministry of Health requested the study presented in this article. The study was done in 2019, at which time the national information campaign was already running. The research questions were:

• What are the information needs of Dutch citizens with LHL regarding organ and tissue donation in general and the new Organ Donation Act in the Netherlands in particular?
• How do Dutch citizens with LHL want to receive information on these topics? Through which types of materials and channels do they want to receive the information?
• What do the results of this study imply for the information strategy towards Dutch citizens with LHL?

The article will end with giving specific suggestions for improving information on organ and tissue donation and the new Organ Donation Act.

## Materials and Methods

Given the explorative character of the subject and the possible difficulties of the target group with reading and writing, a qualitative study design was chosen.

### Participants

Respondents were recruited through organisations that target specific subpopulations with LHL (limited reading and writing skills, minor intellectual disabilities, migrants). These organisations were selected because the research institute had previous worked with them and/or because they were partner organisations of the Dutch Alliance for Health literacy. Inclusion criterion was the subjective acknowledgement of the potential participant that (s)he regularly experienced difficulties with accessing and understanding health information (basic levels of health literacy). The organisations gave potential participants easy and understandable information about the study (on the topic of the study, the aim, methods of datacollection and practical aspects), and then asked people whether they would be willing to participate. The aim was to perform three focus groups or—if people would prefer that—individual interviews, with at least 24 participants (≥18 years) in total and/or till data saturation occurred. Data saturation means that no new topics or themes emerged during the interviews, and new interviews added no more insgihts. All respondents received a gift cheque of 15 euro for their participation.

### Data Collection

A topic questionnaire was developed for the interviews and focus groups. Topics were: current level of knowledge and attitude about organ and tissue donation in general and the new Organ Donation Act in specific. Information needs were discussed, as well as preferred types of materials and channels for receiving that information. Also some existing materials on the new Organ Donation Act (an animated movie on the website www.donorregister.nl, television commercials and a leaflet that was distributed to every household in the Netherlands) were evaluated. The interviews and focus groups started with an explanation of the study purpose. All participants understood the specific research information and consented to participate in the study. Permission was asked and given to record the session and to use anonymized quotes from the interviews and focus groups. Data collection took place in July and August 2019.

### Data Analysis

The (group and individual) interviews were audio-recorded, transcribed verbatim, and anonymized. Each transcript was coded by the researchers, using the research questions and topics as a general framework for thematic analysis. All transcripts and codes were discussed in the research team. Since there were no systematic differences in the topics that emerged from the focus groups and the individual interviews, we analysed them together and combined the information in the results.

### Ethical Approval

This study does not fall within the scope of the Dutch Medical Research Involving Human Subjects Act and therefore does not require ethical approval. All respondents participated on a voluntary basis. They gave informed consent to use their answers for scientific research.

## Results

### Participants

In total, 30 respondents (15 men and 15 women; age 18–65) volunteered to participate in the study. Four focus groups were held in different locations (with 3, 4, 6, and 11 participants respectively). Six respondents were unable or did not want to travel and were individually interviewed at their home. Two focus groups (N = 7) and all individually interviewed persons (N = 6) had difficulties with reading and writing and/or understanding health information. One group were migrants who—despite living in the Netherlands for several years—experienced additional problems with reading and understanding Dutch language (N = 11). One focus group were participants with minor intellectual disabilities (N = 6). At the end of the data collection, data saturation occurred and no new themes were identified. In the analysis phase, no specific differences between the different groups of respondents emerged, therefore we decided to present the data for all respondents with LHL together.

### Knowledge Level and Attitude Regarding the Organ Donation Act

Many respondents—about a third–had not heard at all of the new Organ Donation Act. Those who were aware, lacked basic knowledge. Specifically, they did not really know what it entailed nor what was required of them. Most often they had seen or heard a television or radio commercial, but did not actively seek additional information. The different options were unclear for most respondents and they were not actively thinking about which option would match their opinions and sentiments best. If they reflected on the choice, they were inclined to choose option 1 (to be a donor), as they felt more or less obliged to do so. If anything, thinking about death and organ donation made people anxious and uncertain.

“*And it is also a little bit scary. I used to think if I register, I will be dead by next week…. Or suppose I am not complete dead and they are cutting in me?” (Female).*

Some respondents reported low levels of support for opt-out legislation; they felt angry, because they felt forced by the government to make a decision. Some respondents mentioned that this kind of law was unfair to people with limited (health) literacy skills, since if you do not understand what you have to do and don’t register your personal choice, you will automatically become a donor.

*“People [with LHL] are fooled by wrong links on websites. They are vulnerable and hardly dare to use the computer. Also because they don’t understand everything, they can easily be pushed into making a choice which they actually don’t support.”(Male).*

In general, most respondents believed that they lacked specific and practical information to make an informed personal decision.

### Information Needs

People with LHL expressed a desire for additional information on several key themes. The five main topics identified were:

(1) Choice options and the differences between them: whether it is an obligation to become an organ donor, what the choice options are and whether you can change your decision at a later point in time;
(2) Eligibility: if, on the basis of health or lifestyle, someone is suitable as a donor (e.g., when you smoke or when you have a chronic disease), and whether one’s religion allows you to be a donor or not;

*“I have diabetes, and, yes, I do not know about the parts in my body … how good they still are.”(Male).*

*“I am a religious woman. We talked about it in the community centre. Some people had difficulties with it. They wonder whether God would approve if I would donate my organs. They think that God would not want that.” (Female).*

(3) Role of the partner and/or family: respondents want to know what happens if multiple relatives don’t agree (in case of option 3), or when you are registered as a donor and your partner or relatives oppose to that;

*“But your, father or mother, … they can say no, for example. Because you are automatically in [the register], if you do nothing. And then you are automatically a donor, when you die. Then your parents can say, she doesn’t want it, even though she is in it. Right? Or not? How about that?” (female).*

(4) Impact on quality of care: some respondents expressed fears around medical mistrust. For example, they expressed concerns that the care they receive will be negatively affected;

*“If I now say that I will be a donor, are they still going to help me well when I am ill, or do they think, she can better be dead, because then we have organs again.’ (Female).*

(5) Process of organ donation: some respondents want to know what actually happens after your death, how much time there is before you are taken away, about the medical procedure itself and how the process impacts the funeral and its preparations.

*"I want to know if, when I say yes, what will happen to my body? What happens then? Can you still say goodbye to someone in a decent way? (Female).*

*“Somebody then scared me. They said that when you are dead, it takes very long before the family gets your body back. Because it is taken away. Because everything has to be taken out. I don’t want that. Then I renounced it.” (Female).*

Furthermore most respondents requested practical help in registering their choice through the website, since they have difficulties with or are unable to use computers.

*“I prefer a little bit the old-fashioned, really filling out on paper. If I really have to, I could do it [digitally]. But I feel that I often have to be helped by someone, together, and watching with me.”(Male).*

Also help in retrieving and filling out the paper form, which you can also use for registering, was desired.

### Evaluation of Current Information Materials

The content of the information currently used was generally considered too difficult and too abstract to make an informed decision whether or not to be an organ donor. Three materials were specifically discussed with the respondents: an animated movie, television commercials and a folder that was distributed to every household in the Netherlands. In general, the respondents expressed a preference for information in a spoken form, in short movies or animations.

*“A video is clear, because it clearly shows what is exactly happening… A paper, I would read but not understand at all. The letter is too much text.” (Female).*

Nevertheless, only two of the participants had seen the animated movie before the focus group session, even though it was broadly distributed by the government. Main advantages of the animated movie were that the information was spoken (not too quick), that much information was presented in a clear way and that the website address and phone number, where more information could be obtained, was visible long enough. Main disadvantages were that people were not aware of these movies, and that they were accessible through a website only. Suggestions for improvements were: 1) show examples of “real” people who tell why they decided to choose for one of the options (narratives) and 2) actually show the process of registering, step-by-step, how one should do that, and 3) make the movie available in different languages.

Most respondents mentioned seeing the television commercials. They remembered that famous Dutch persons played a role in it, and were positive of the diversity of characters in the commercials. They considered the commercials funny, and good for general awareness since television is an accessible medium. However, the main message of the commercials was not clear. They were too short, and didn’t provide enough background information on the registration process. Information was not repeated and also the website address and telephone number were only shown briefly.

A leaflet in general was considered useful because it contains all relevant information and could be used as a reference. However, the content and language level of the leaflet that was distributed in all Dutch households was considered much too difficult: too much text, too long sentences. Words like organs, donation and donor register were difficult to comprehend.

*“If you would ask me “what does ‘donor’ exactly mean?” Than I would not know that at all. I cannot explain what it is.” (Female).*

In general, the leaflet was not readable for most respondents, especially those with limited reading skills. Respondents suggested to use more pictures and animations and less text in the leaflet. The fact that a logo of the Dutch government/Ministry of Health was clearly printed on the front of the leaflet raised ambivalent reactions. Though it was considered positive that you know who the sender of the leaflet is, most of the respondents back off if they get mail from the government, either because they know from personal experience that it will be difficult to comprehend, but also because mail from the government usually is bad news (e.g., taxes).

### Preferred Information Materials and Channels

Most respondents suggested to make all materials on organ donation and the new law easier to comprehend in general, so that they could be used universally and no specific materials for people with LHL would have to be developed. However, since they do have additional information needs, some extra communication strategies seem warranted. In general, the respondents preferred simple movies and narratives of other people. An easy to read leaflet would be appreciated. Some respondents also use the Internet as a source of information, but for others this is too difficult.

The respondents stressed the importance of actual personal support, in order to understand and personalise the information, discuss the options and help with the actual registration. They suggested the involvement of organisations and professionals within their personal network, e.g., organisations that support people with intellectual disabilities, neighbourhood teams, schools, health care organisations and providers. Also the social network (family, friends) was considered very important to discuss this complicated topic with.

## Discussion

### Main Findings

Many of the people with LHL in this study had not heard at all of the new Organ Donation Act that was to be implemented in the Netherlands. Those who were aware (usually through television or radio commercials) did not know what it entailed nor what was required of them. Participants lacked the information they needed to make an informed personal decision. They expressed a need for more specific information on organ donation and what the new law entailed. The five key themes that emerged were: 1) choice options and the differences between them, 2) eligibility, 3) role of the partner and/or family, 4) impact on quality of care and 5) the process of organ donation. Furthermore they expressed a need for practical help in registering their choice.

Current information on the new Dutch law on organ donation was generally considered too difficult and abstract. The importance of actual personal support was stressed, in order to understand and personalise the information, discuss the options and help with the actual registration. The respondents suggested the involvement of organisations and professionals they already have contact with, like their GP or a social worker. Also the social network (family, friends) was regarded an important source for help and support.

This study shows that people with LHL have more difficulties with understanding and applying health information and making informed choices, which has been demonstrated in many other studies and health contexts (e.g., cancer screening, screening for Down in pregnant women, health decisions in general) [10–16]. People with LHL need information that is easy to understand and relatable. Written information is often considered too difficult to comprehend, and generally too abstract. They are interested in experiences of others and narratives [17]. They also express a need for more practical information, e.g., a step by step explanation of what is to be done. Furthermore, they require more personal support in making health related choices and decisions. Our study confirms these results in the context of organ donation.

### Strengths and Limitations of the Study

To our knowledge, this is the first study on the information needs and preferred information channels of people with LHL regarding organ donation. It is a strength of this qualitative study that representatives from this target group could express their own needs and preferences, since they are often underrepresented in quantitative scientific studies. The participants in our study were recruited through different organisations, each with a focus on a specific subpopulation. This led to a representation of various subgroups of people with LHL in our study (people with reading and writing difficulties, migrants, people with minor intellectual disabilities). Though this is a qualitative study with only 30 participants, we think this diversity and the fact that saturation occurred makes the results generalizable to the larger group of people with LHL. The subjects selected acknowledged their difficulties with regard to understanding health information. This might generate some bias in the sample, as people with LHL who do not acknowledge these difficulties might have different needs. Another limitation of our study is that not in all focus groups, the current information materials were systematically discussed, due to time constraints and different discussion priorities of the participants. However, where it was done, reactions and answers all pointed in the same direction.

### Implications for the Information Strategy for People With LHL

Some of the current materials were considered useful, but should be more accessible (e.g., the animated movie) or adapted to the reading level and information needs of people with LHL (e.g., the information leaflet). Including less text, long sentences and difficult, abstract words, and more specific information on the topics that were mentioned by the respondents. Co-creation and pre-testing such a leaflet with representatives from the target group is recommended. As information in spoken form was preferred, the respondents in our study also suggested to make special movies for specific target groups together with them, and distribute them through regular channels of the organisations they already have contact with and access to. All these materials would focus on knowledge, one of the cognitive attributes of health literacy [6]. For actual behaviour to take place, attention for the other (behavioural/operational and affective/conative) attributes is also important [6, 7]. The respondents also stressed that they require practical information and personal support. People with LHL often also have limited computer skills, so seeking information on the Internet, registering one’s choice through a website or finding information on how to get a paper form is a special challenge with which they need support. People also need support in order to tailor the general information on organ donation to their personal situation. This can also help in reducing the anxiety and uncertainty that was expressed by many people in our study. This support can either be provided by professionals they already know (e.g., teachers, case workers or health care providers), by people from their own social network and/or by volunteers who are present in community centres where materials are distributed. It is important that the professionals actually coach the person with LHL in making the decision that best fits their situation and wishes, by providing understandable information on all options (and not only the one they would consider best) and through methods used in shared decision making, such as value elicitation. Since people with LHL heavily rely on persons they trust, it is important to remain neutral with respect to the options and refrain from “advising” in a certain direction.

## Conclusion

People with LHL need specific information to make an informed decision on organ donation. This information should be accessible and easy to understand. Current standard materials are too difficult and abstract. Furthermore, they require personal support to tailor general information to their personal situation, and practical help to actually register their choice.

## Data Availability

The raw data supporting the conclusions of this article will be made available by the authors, without undue reservation.
